# Changes of the serum properties and its effect on the endothelial cells restoration in patients with chronic venous disease treated with sulodexide

**DOI:** 10.1016/j.jvsv.2024.101941

**Published:** 2024-06-28

**Authors:** Adam Zieliński, Katarzyna Jasińska-Sumińska, Andrzej Bręborowicz, Katarzyna Kowalska, Maciej Zabel, Teresa Wysocka, Raouf A. Khalil, Joseph D. Raffetto, Tomasz Urbanek

**Affiliations:** a Section of Surgery, Vascular Surgery and Phlebology, doktorA Medical Center, Warsaw, Poland; b Department of Pathophysiology, Poznan University of Medical Sciences, Poznań, Poland; c Department of Anatomy and Histology, University of Zielona Góra, Zielona Góra, Poland; d Department of Histology and Embryology, Poznan University of Medical Sciences, Poznań, Poland; e Vascular Surgery Research Laboratories, Division of Vascular and Endovascular Surgery, Brigham and Women’s Hospital, and Harvard Medical School, Boston, MA; f VA Boston Healthcare System, Harvard Medical School, Brigham and Women’s Hospital, Boston, MA; g Department of General Surgery, Vascular Surgery, Angiology and Phlebology, Medical University of Silesia, Katowice, Poland

**Keywords:** Chronic venous disease, Varicose veins, Inflammation, Endothelium, Glycocalyx, Sulodexide

## Abstract

**Objective:**

Inflammation and endothelial dysfunction are important venous changes in patients with chronic venous disease (CVD). The use of the venoactive drugs remains an important treatment modality for patients with CVD, reducing the severity of the CVD-related symptoms and swelling but also reducing inflammation and protecting endothelial cells. In this research, the effects of the serum obtained from patients with CVD before and after sulodexide treatment were evaluated for in vivo and in vitro inflammatory markers and endothelial cell function.

**Methods:**

Inflammatory markers (IL-6, matrix metalloproteinase-9 [MMP-9], vascular cell adhesion molecule-1 [VCAM-1], and von Willebrand factor [vWF]) from the incompetent great saphenous veins (GSVs) and from the systemic venous circulation were studied in 10 patients with CVD (C2s) before and after 2 months of sulodexide (2 × 500 lipasemic units/d) therapy. Serum obtained from the vein blood before and after sulodexide treatment was evaluated for in vitro cultured human umbilical vein endothelial cell function.

**Results:**

The serum collected from lower leg incompetent GSVs had significantly elevated levels of VCAM-1 (+29%, *P* < .001) compared with the serum from the systemic circulation. Endothelial cells exposed to the serum from the incompetent lower leg veins of the untreated CVD patients demonstrated higher stimulated synthesis of MMP-9 (+17%, *P* < .01), as well as increased markers of senescence (prolongation of population doubling time, β-galactosidase activity, and expression of p21 and p53 genes). CVD serum-induced senescent endothelial cells had a higher expression of genes regulating IL-6, MMP-9, VCAM-1, and vWF synthesis. The overall proinflammatory effect on endothelial cells by the serum collected from the incompetent GSVs was stronger as compared with the serum from the systemic circulation. Serum collected from the veins after sulodexide treatment caused lower levels of endothelial cell inflammatory markers as well as respective gene expression than serum obtained at the beginning of the study (before sulodexide treatment). Sulodexide application also reduced the inflammatory secretory activity of the senescent endothelial cells. Sulodexide treatment resulted in the decrease of the majority of the studied inflammatory parameters in both lower limb incompetent vein and systemic blood.

**Conclusions:**

In patients with CVD, there are significant differences between circulating inflammatory markers analyzed from the lower leg incompetent GSV segments compared with the systemic circulation, indicating a higher inflammatory condition in CVD. Treatment with sulodexide reduces the proinflammatory and endothelial cell activation properties of the serum from patients with CVD.

**Clinical Relevance:**

The study documented the significant proinflammatory human vascular endothelial cell activation when exposed to the serum collected from the varicose veins as compared with the serum from the systemic circulation in patients with chronic venous disease (CVD). The inflammatory marker expression, endothelial dysfunction, and endothelial cell senescence transformation can be successfully controlled and downregulated by patients’ exposure to the glycosaminoglycan (sulodexide) treatment. Further studies are needed to confirm if glycosaminoglycan application can prevent further CVD clinical progression due to potential CVD-related pathological processes’ modulation and their downregulation.

**Article Highlights**

• **Type of Research:** Experimental research
• **Key Findings:** Endothelial cells exposed to the serum from the incompetent saphenous veins demonstrated higher synthesis of matrix metalloproteinase-9 and increased markers of cell senescence when comparing with the effect of the serum from systemic circulation. Sulodexide lowers the endothelial inflammatory marker expressions and reduces the inflammatory secretory activity of the senescent endothelial cells.
• **Take Home Message:** The proinflammatory and endothelial cell activation properties of the serum obtained from the patients with chronic venous disease can be successfully controlled by sulodexide.

Chronic venous disease (CVD) is a prevalent disease, which creates significant medical problems and financial burden to the health care system and causes a reduction in the quality of life.^1^ Venous hypertension may result in an elevated inflammatory state and endothelial cell (EC) dysfunction in the macro- and microcirculation.^2^ After the activation of inflammatory mediators, the expression of growth factors and matrix metalloproteinases (MMPs) leads to the disorganization and degeneration of the vein wall.^2^, ^3^, ^4^, ^5^, ^6^, ^7^, ^8^

Sulodexide, which is used in peripheral vascular disease treatment (including CVD), is a purified glycosaminoglycan and contains a mixture of 80% of fast moving heparin and 20% of dermatan sulfate. Sulodexide has a wide range of biologic effects, such as anti-inflammatory, antioxidant, and antithrombotic properties, as well as the protective effect toward the venous ECs.^9^, ^10^, ^11^, ^12^ One of the activities related to the pleiotropic effect of sulodexide is endothelial glycocalyx restoration.^11^, ^12^, ^13^ The disruption of the glycocalyx and its function as well as structure impairment result in EC activation and detrimental changes in endothelial antithrombotic and anti-inflammatory integrity.^11^, ^12^, ^13^, ^14^, ^15^, ^16^, ^17^, ^18^, ^19^, ^20^, ^21^

According to the current CVD pathogenesis theory, there are predisposing and environmental factors, glycocalyx injury, and endothelial dysfunction resulting in an inflammatory state with venous wall and valve dysfunction.^1^^,^^3^ Previous studies in patients with CVD demonstrated intravascular inflammation having a different quality and intensity in the blood present in the peripheral varicose veins and systemic circulation. Studies have determined that blood in the varicose veins had a higher concentration of IL-6, C-reactive protein, von Willebrand factor (vWF), and D-dimers than blood obtained from the nonvaricose veins in the lower extremity.^22^ This finding may indicate that venoactive drug treatment for CVD may have different responses based on affected varicose veins and unaffected normal veins. According to the studies performed, sulodexide reduces the intravascular systemic inflammation parameters in patients with CVD,^23^^,^^24^ but it is yet to be determined if sulodexide has a differential effect on inflammatory molecules in varicose veins and nonvaricose veins. In this study, we compared the levels of the inflammatory markers in the incompetent great saphenous veins (GSVs) and the systemic venous circulation in patients with CVD, and evaluated how sulodexide treatment would affect the regional and systemic inflammatory reaction parameters. Our second objective was to study human EC inflammation and senescence transformation when subjected to the serum from untreated and sulodexide-treated CVD patients.

## Methods

### Patients, blood, and tissue collection

The study was performed on a group of 10 symptomatic varicose vein (C2s) patients (mean age: 41 ± 16 years) selected from the 34 consecutive C2 patients with GSV incompetence screened in the first author center. In all the patients, both leg varicose veins were present. In the venous ultrasound Doppler examination, in all cases, bilateral GSV incompetence (reflux >0.5 seconds) from the groin to the upper one-third of the calf with varicose veins of the saphenous origin was found. None of the patients presented clinically with leg edema, and in all the patients, the CVD symptoms were confirmed (leg heaviness: 90%, leg pain: 70%, and cramps: 40%). To obtain the homogeneous study group, the subjects with concomitant anterior accessory, perforator, and small saphenous as well as deep vein incompetence were excluded. Among other exclusion criteria the following were specified: previous deep or superficial vein thrombosis, previous invasive treatment of the CVD, statin, anticoagulant, antiplatelet or venoactive drug administration currently or in the past, presence of diabetes, renal failure, peripheral arterial occlusive disease, active infection, any known inflammatory conditions, and the use of compression stocking. In all the cases, the qualification for the endovenous treatment was based on the presence the GSV incompetence-related varicose veins together with the presence of CVD symptoms. All the patients were referred for endovenous laser ablation (EVLA) of the GSV followed by the second EVLA in the contralateral leg during a separate (delayed) admission.

On the day of the first EVLA, blood samples were collected from a peripheral vein near the cubital fossa as representative of the systemic venous circulation and from the incompetent GSV trunk at the upper third of the calf in the leg contralateral to the lower extremity undergoing endovenous treatment during the same visit (this leg was scheduled to the EVLA in a second, separate term). In seven patients, the vein access was obtained percutaneously, and in three patients, venous access was obtained by direct surgical exposure of the GSV. In this second group (with open vein access), the GSV wall microbiopsy was taken comprising a specimen that was used for the glycocalyx thickness evaluation by transmission electron microscopy (TEM). Immediately after vein wall excision, the samples were fixed by placing them in a solution of 2.5% glutaraldehyde buffered with 0.1 M phosphate solution and transported to the TEM lab. EVLA procedures were performed in accordance with standard practice in this type of treatment under tumescent anesthesia. All patients received thromboprophylaxis with 40 mg of enoxaparin started 12 hours before EVLA and for a total of 5 days. A 1470-nm diode laser (Biolitec) with radial fibers was used. After EVLA, the patient received a class II compression stocking for 4 weeks after surgery (on the operated leg only). On day 6 (24 hours after the last prophylactic low molecular weight heparin dose), all patients started sulodexide administration: two capsules of 250 lipasemic units (LSU) twice daily for the first 10 days and then 1 capsule of 250 LSU twice daily for the following 76 to 98 (median 80) days. The consecutive delayed contralateral GSV EVLA was performed in the second leg in a period of 78 to 110 days after the first surgery. Patients continued sulodexide administration till the day of the procedure (if performed later than 78 days after primary EVLA). The course of the procedure was the same as during the first EVLA treatment. On the day of the second (contralateral) EVLA, a blood sample was collected from the cubital fossa vein and from the operated GSV trunk in the upper calf at the beginning of the endovenous procedure (both blood samples collected from the patients during the study period were collected from the same leg incompetent GSVs). Regarding the second EVLA venous access, in the same seven patients, the venous access was obtained percutaneously, and in the three other patients, with previous surgical GSV access, direct surgical access was repeated and GSV wall biopsies were obtained for TEM analysis. The control vein specimens for the TEM were vein samples obtained from the GSV removed during lower limb (LOL) lipoma resection from a healthy 38-year-old male patient without CVD.

### Collected patient serum, culture media conditions for HUVECs

In the collected serum samples, concentrations of IL-6, MMP-9, vWF, and vascular cell adhesion molecule-1 (VCAM-1) were measured with standard ELISA kits (R&D Systems). In addition, the effect of serum samples on human umbilical vein EC (HUVEC) in vitro culture was evaluated. The study was performed on HUVECs (Life Technologies Corp) cultured in medium M200 supplemented with 2% fetal bovine serum, hydrocortisone 1 μg/mL, heparin 10 μg/mL, human epidermal growth factor 10 ng/mL, and basic fibroblast growth factor 3 ng/mL. Experiments were performed on cell monolayers. In the first experimental part, the effect of the collected serum samples on the secretory activity of the ECs was studied. Cell monolayers in 24-well plates were incubated for 24 hours with the following five culture media conditions:

• Medium M200 supplemented with 20% control serum/Control/ (the control serum was collected from the same age healthy individuals without any inflammatory conditions and without known arterial as well as venous disorders)
• Medium M200 supplemented with 20% serum from the upper limb (UPL) of patients with CVD collected at the beginning of the study (before sulodexide treatment)/UPL Start/
• Medium M200 supplemented with 20% serum from the UPL of patients with CVD collected at the end of the study (after sulodexide treatment)/UPL End/
• Medium M200 supplemented with 20% serum from the LOL of patients with CVD collected at the beginning of the study (before sulodexide treatment)/LOL Start/
• Medium M200 supplemented with 20% serum from the LOL of patients with CVD collected at the end of the study (after sulodexide treatment)/LOL End/

At the end of the 24-hour incubation period (which, according to our previous experiments, induced the maximum acute effect in EC functional properties with no reduction in their viability), the media was removed from the wells and replaced in all groups with serum-free medium to evaluate the secretory activity of the cells after 24 hours of incubation. At the end of incubation, supernatants were collected for the measurement of IL-6, MMP-9, VCAM-1, and wWF. Cells from the wells were removed with trypsin 0.05%-ethylenediaminetetraacetic acid 0.02% solution and counted in a hemocytometer. Secretory activity of the ECs was expressed per number of cells.

### Effect of the studied sera on cellular senescence

The senescence of the ECs was induced by 10 repeated passages at 5-day intervals and in the presence of medium supplemented with the control serum or with pooled serum from each experimental group listed above. Cells were seeded in 25 cm^2^ flasks at a density of 5 × 10^3^/cm^2^ and cultured for 5 days. At the end of that time, cells were harvested from the culture flask, counted in a hemocytometer, and again reseeded into the culture flask. At each subcultivation, the harvested cells from the flask were counted in a hemocytometer, and their population doubling time (PDT) was calculated according to the following formula: PDT = ln2/[ln(*N*/*N*_0_)/*t*], where *N* is the number of cells harvested at the end of the 5 days’ culture, *N*_0_ is the number of cells seeded into the flask, and *t* is the time duration of the culture.

At the beginning and at the end of the experiment, cells were seeded into 24-well plates for the evaluation of their secretory activity as described above. Cells were also cultured in Labtec wells for staining for the presence of β-galactosidase (β-galactosidase staining kit; Mirus), which was used as an index of the cellular senescence. Cells were also cultured in six-well plates for RNA isolation and evaluation of the gene expression.

### Gene expression analysis

Total RNA was isolated from the cells’ monolayer using a commercially available kit (Thermo Fisher Scientific). Quantitative reverse transcription-polymerase chain reaction was started by amplifying cDNA with the Bio-Rad iScript cDNA synthesis kit (Bio-Rad) according to the kit manufacture instructions. Expression of the genes was analyzed by quantitative polymerase chain reaction with the FastStart SYBR Green Master Mix (Roche). Expression of the following genes was studied: cyclin-dependent kinase inhibitor 1 [p21], tumor protein p53 [p53], IL-6, vWF, MMP-9, and VCAM-1 and normalized with the housekeeping genes: glyceraldehyde-3-phosphate dehydrogenase and hypoxanthine phosphoribosyltransferase 1.^25^ The list of the primers is presented in Supplementary Table I (online only).

### Effects of sulodexide on senescent endothelial cells

In the next set of experiments, ECs reaching senescence by repeated passages were evaluated in medium supplemented with control serum or pooled serum collected from the UPL and LOL at the beginning of the study. The treated senescent ECs were seeded into 24-well culture plates and grown to monolayers. The secretory activity of the ECs was evaluated in standard culture medium with or without sulodexide 0.5 LSU/mL treatment.

### Glycocalyx assessment

In the three patients with GSV bilateral biopsy, the glycocalyx layer of the treated veins was assessed in the specimens. To enhance the electron density of polysaccharides included in the glycocalyx layer, the specimens were stained with ruthenium red. This cationic dye connects with the negatively charged glycocalyx increasing the electron contrast.^26^^,^^27^ The specimens were examined by TEM (model JEM 1010; Jeol) equipped with the Megaview G2 camera and automatic picture imaging system (Olympus). The measurements were performed in the perpendicular axis to the cell membranes, and the width of the glycocalyx was established as a distance from double-layered cell membranes toward the lumen of the vessel. Several measurements of the glycocalyx layer were performed in each specimen for detailed sections. The results were compared with the control GSV specimens. The research project was approved by the ethical committee, and informed consent was obtained from all patients.

### Statistical analysis

Results are presented as mean ± standard deviation. Statistical analysis was performed with the Wilcoxon test or analysis of variance with the Newman-Keuls post hoc test. A *P* value of less than .05 was considered statistically significant. In the result description, except graphical presentation, the percentage changes reflecting the changes in the concentration of the studied molecules per volume or the number of cells were also used.

## Results

### Serum levels of the investigated parameters (in vivo evaluation)

Comparing the initial levels of the studied parameters (IL-6, MMP-9, VCAM-1, and vWF) in the serum from the incompetent GSVs and in the serum from the systemic circulation, a significantly higher level of VCAM-1 (+29%, *P* < .001) in the blood serum obtained from lower legs was observed.

**Fig 1:**
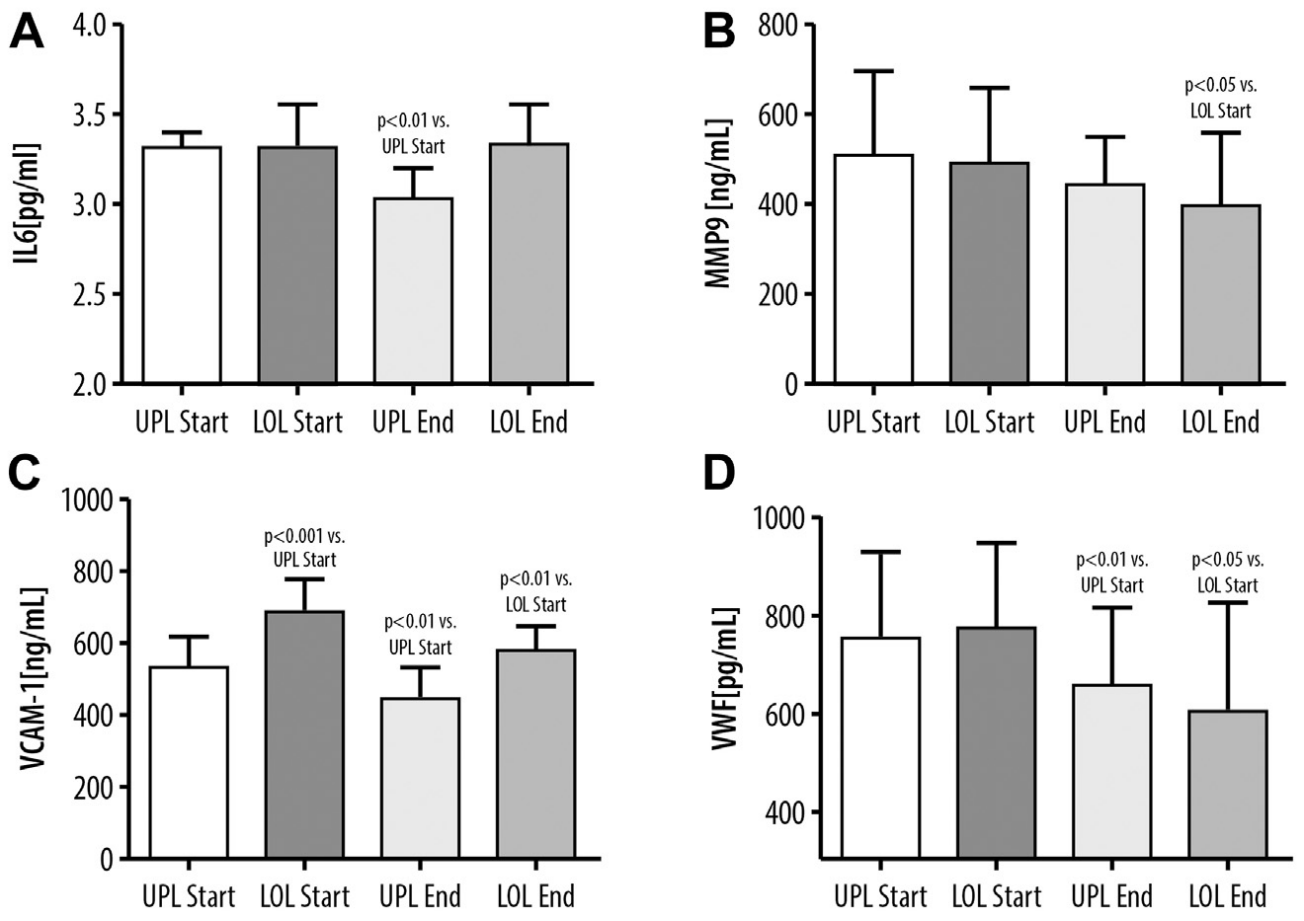
Blood levels of IL-6 **(A)**, matrix metalloproteinase-9 (*MMP-9*) **(B)**, vascular cell adhesion molecule-1 (*VCAM-1*) **(C)**, and von Willebrand factor (*vWF*) **(D)** collected from the upper limb (*UPL*) veins or lower limb (*LOL*) incompetent veins before sulodexide treatment (*Start*) or at the end of the experiment after sulodexide treatment (*End*).

Sulodexide treatment resulted in a decrease of the majority of the measured inflammatory and procoagulant factors. The VCAM-1 level was reduced in serum obtained from the UPL (–15%, *P* < .01) and LOL (–15%, *P* < .01) after sulodexide treatment. Similar findings were observed with vWF levels, which were also reduced in both the UPL and LOL, demonstrating a reduction of 13% (*P* < .01) in the UPL blood serum and 22% (*P* < .05) in the serum obtained from the LOL after sulodexide treatment. Concerning the other studied parameters, the reduction of the MMP-9 concentration after sulodexide treatment was found only in the serum from the LOL incompetent veins, and the IL-6 level remained unchanged in the serum from the LOL and decreased in the serum from the UPL (–10%, *P* < .01) (Fig 1).

### Synthesis of IL-6, MMP-9, VCAM-1, and vWF in EC cultures exposed to control medium or to medium supplemented with the serum from the study patients

**Fig 2:**
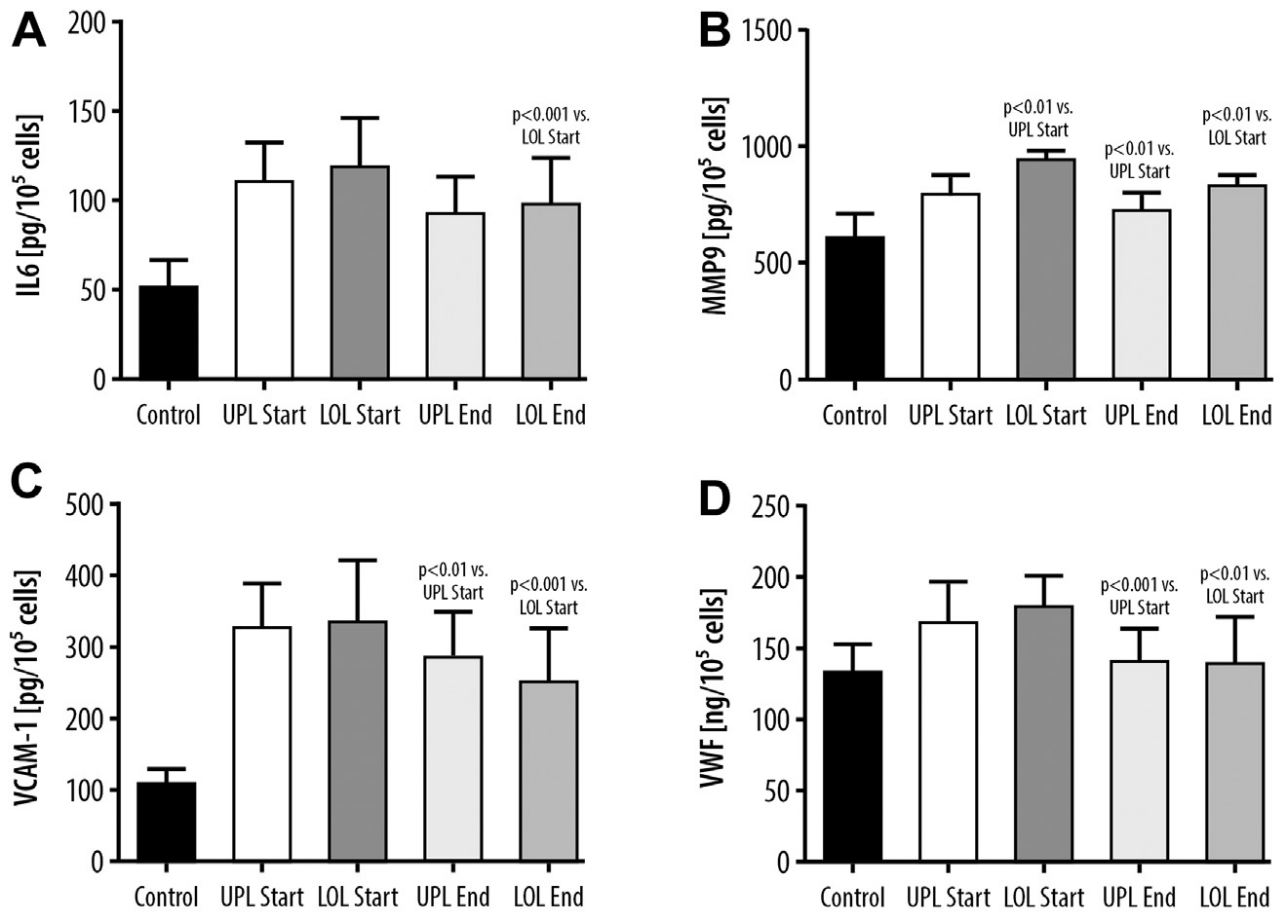
Synthesis of IL-6 **(A)**, matrix metalloproteinase-9 (*MMP-9*) **(B)**, vascular cell adhesion molecule-1 (*VCAM-1*) **(C)**, and von Willebrand factor (*vWF*) **(D)** in endothelial cells exposed to control medium or to medium supplemented with the serum from the upper limb (*UPL*) or the lower limb (*LOL*) collected before sulodexide treatment (*Start*) or at the end of the experiment after sulodexide treatment (*End*).

Exposure of the EC to the different serum conditions (UPL Start, UPL End, LOL Start, LOL End), collected from patients with CVD at the beginning and at the end of the study, caused a significant increase in cellular activation and secretory activity of IL-6, MMP-9, VCAM-1, and vWF as compared with treatment with the control serum (Fig 2). Comparing the effect of the serum obtained from the upper extremity veins and incompetent GSVs at the start and before sulodexide treatment, we demonstrated an increased synthesis of MMP-9 in the ECs when using the serum from the LOL veins (+17%, *P* < .01). No significant differences in the IL-6, VCAM-1, and vWF levels were observed in the ECs exposed to UPL Start and LOL Start serum conditions between the UPL and LOL.

Comparing the levels of the above parameters after exposure of the ECs to the serum samples obtained before and after sulodexide treatment, there was a significant reduction in the concentration of all inflammatory markers after incubation with the serum obtained from the incompetent GSVs at the end of sulodexide treatment (IL-6 concentration reduction: –18%, *P* < .001; MMP-9: –12%, *P* < .01; VCAM: –24%, *P* < .001; vWF: –24%, *P* < .01). Concerning systemic circulation parameters, EC incubation with the serum obtained from the UPL after sulodexide treatment led to the reduced synthesis of MMP-9 (–10%, *P* < .01), VCAM-1 (–13%, *P* < .01), and vWF (–16%, *P* < .001). No changes in IL-6 concentration were observed when using the serum from UPL superficial veins after sulodexide treatment (Fig 2).

### Endothelial cell senescence, IL-6, MMP-9, VCAM-1, and vWF gene expression, influence of sulodexide treatment

**Fig 3:**
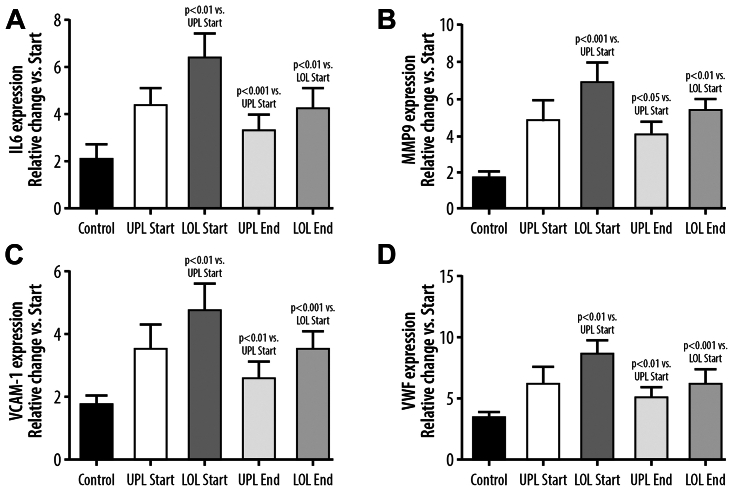
Changes, as compared with the beginning of the study, in expression of genes regulating synthesis of IL-6 **(A)**, matrix metalloproteinase-9 (*MMP-9*) **(B)**, vascular cell adhesion molecule-1 (*VCAM-1*) **(C)**, and von Willebrand factor (*vWF*) **(D)** in endothelial cells undergoing replicative senescence in the presence of the control serum (*Control*) or the serum collected from the upper limb (*UPL*) or lower limb (*LOL*) obtained before sulodexide treatment (*Start*) or at the end of the experiment after sulodexide treatment (*End*).

After 10 passages, there were signs of EC senescence in all experimental groups. Senescent ECs exposed to the serum from patients with CVD had higher expression of genes regulating IL-6, MMP-9, VCAM-1, and vWF synthesis. The effect of the serum collected from the varicose veins before the start of the sulodexide was stronger than the effect of the serum from the systemic circulation (Fig 3). Treatment with sulodexide resulted in a statistically significant reduction of the above-mentioned gene expression when the serum from either the UPL or LOL veins was used (Fig 3).

**Fig 4:**
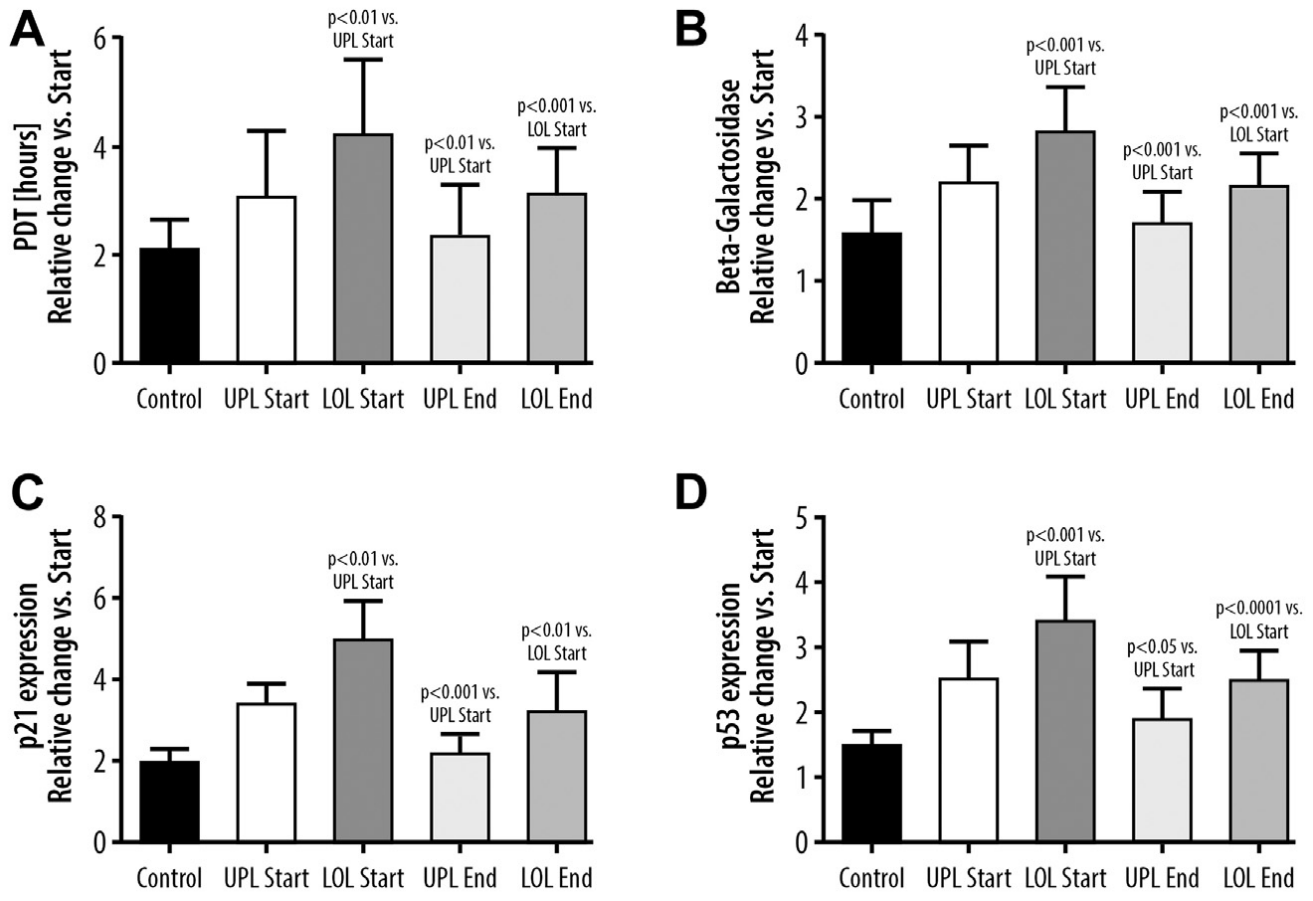
Changes, as compared with the beginning of the experiment, in population doubling time (*PDT*) **(A)**, β-galactosidase **(B)**, expression of p21 gene **(C)** and p53 gene **(D)** in endothelial cells undergoing replicative senescence in the presence of the control serum (*Control*) or the serum collected from the upper limb (*UPL*) or the lower limb (*LOL*) obtained before sulodexide treatment (*Start*) or at the end of the experiment after sulodexide treatment (*End*).

The senescence of the ECs corresponded with the prolongation of the PDT, and increased staining for β-galactosidase was present for both control and CVD patient serum-exposed cell cultures. In the control ECs, the PDT at the beginning was 90 ± 17 hours, which increased to 181 ± 18 hours after 10 passages (*P* < .001). Prolongation of the PDT of the ECs exposed to the serum of patients with CVD before sulodexide treatment was significantly higher as compared with control (*P* < .001). After 10 passages, the staining for β-galactosidase increased in the control group by 155%, *P* < .001, and this effect was greater in the studied EC cultures exposed to the serum from patients with CVD not treated with sulodexide (*P* < .001) (Fig 4). Expression of p21 and p53 genes was increased in the senescent cells in the control group by 140% and 150%, *P* < .001, respectively, and that the effect was stronger in cells treated with the CVD-tested sera. Both prolongation of PDT and β-galactosidase staining were significantly higher in the ECs exposed to the serum from the incompetent GSVs than to the serum from the blood taken from systemic circulation. Also, the expression of p21 and p53 genes was significantly higher when the serum from the LOL veins was used (Fig 4). The results obtained with the use of the serum from the patients after the course of the sulodexide therapy showed a decrease of the PDT and β-galactosidase staining in the senescent ECs when using the serum from the incompetent LOL veins as well as the serum from systemic circulation. Moreover, a decrease of p21 and p53 expression was observed when using the serum after sulodexide treatment (using both serum from GSVs and serum obtained from systemic circulation).

**Fig 5:**
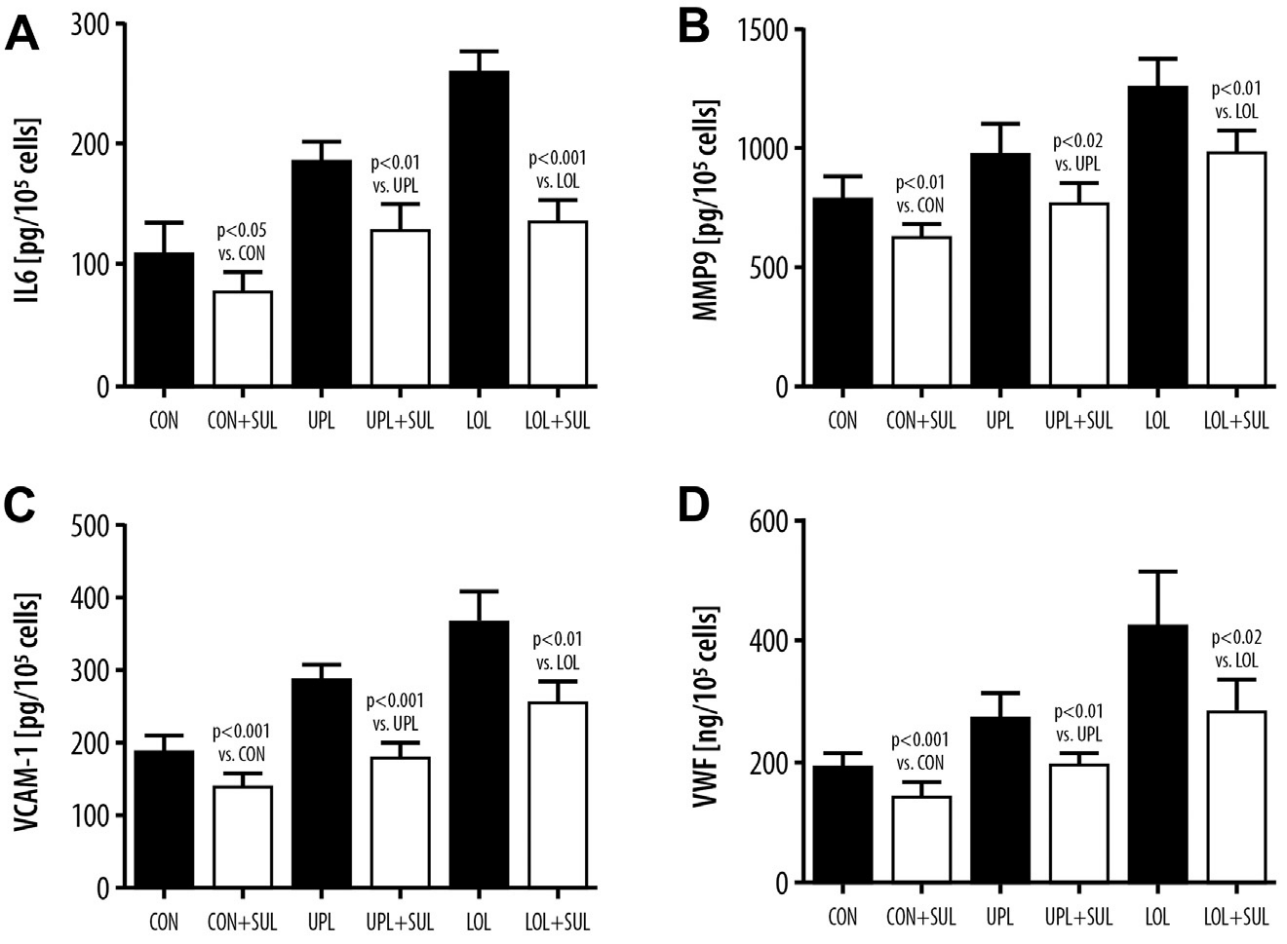
Effect of sulodexide 0.5 lipasemic units/mL (SUL) on secretion of IL-6 **(A)**, matrix metalloproteinase-9 (*MMP-9*) **(B)**, vascular cell adhesion molecule-1 (*VCAM-1*) **(C)**, and von Willebrand factor (*vWF*) **(D)** in the endothelial cells that are senescent by repeated passages in medium supplemented with the control serum (*Control*) or with the serum collected at the beginning of the study (before oral sulodexide treatment) from the upper limb (*UPL*) or the lower limb (*LOL*).

The direct effect of sulodexide on endothelial cultured cell medium was also evaluated. The addition of the sulodexide to culture medium (0.5 LSU/mL) reduced the concentrations of IL-6, MMP-9, VCAM-1, and vWF in senescent EC cultures. The findings were noticed in the EC cultures with control serum as well as in the cultures with the serum collected from the UPL and LOL veins before the sulodexide treatment (Fig 5).

### Transmission electron microscopy

**Fig 6:**
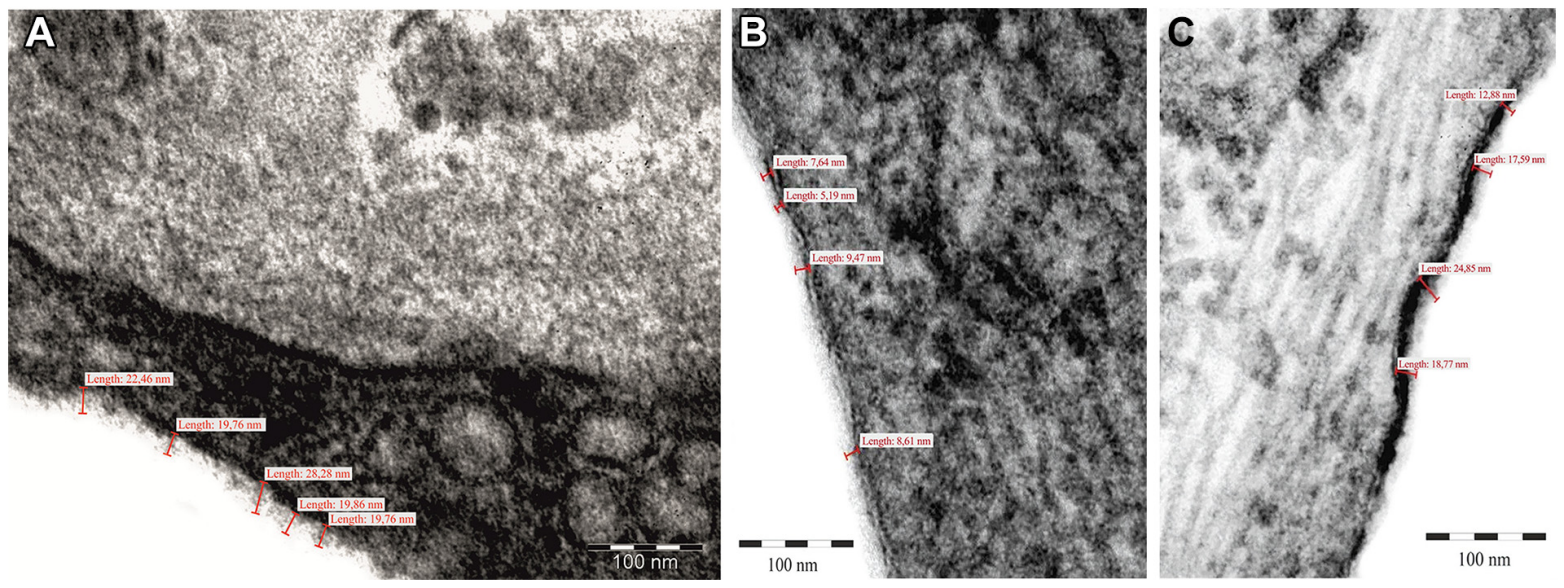
Example of the measurements of glycocalyx thickness in a healthy, competent vein (control) **(A)** and in the vein biopsy specimens collected from the lowest point of an axial reflux in patients with chronic venous disease (CVD) **(B)**, the great saphenous vein (GSV) biopsy taken during the first endovenous laser treatment procedure (*left leg*); **(C)** the biopsy taken from the contralateral limb incompetent saphenous vein during second endovenous laser treatment (*right leg*). The specimen contrasted with ruthenium red from electron microscopy (magnification × 200,000)—thickness of the glycocalyx layer marked.

The vein specimens obtained from the GSV biopsy during the open access to EVLA procedures were used for the assessment of the glycocalyx thickness by TEM. Multiple glycocalyx thickness measurements were performed, and the results in CVD veins were compared with the control healthy vein measurements. The thickness of glycocalyx on the surface of the healthy vein ECs ranged from 19.76 to 28.28 nm (Fig 6, *A*). In patients with CVD, the incompetent GSV glycocalyx thickness ranged between 5.19 and 24.85 nm (Fig 6, *B* and *C*). Due to the lack of the specimens taken from the same veins before and after sulodexide treatment, the objective analysis of the relationship between glycocalyx thickness and sulodexide administration was not possible. However, in the patients with CVD, the vein segments with the variations concerning the glycocalyx layer thickness were found, which suggests glycocalyx abnormalities in this clinical condition, as documented in TEM of the incompetent GSV specimens (Fig 6, *B* and *C*).

## Discussion

This study was performed to determine the differences in inflammatory markers between the upper and lower extremities of patients with CVD. The study also evaluated the effects of untreated and sulodexide-treated serum from patients with CVD on inflammatory expression, endothelial function, and senescence transformation on human ECs. The overall inflammatory effects on ECs of the serum collected directly from the incompetent GSVs were stronger as compared with the serum from the systemic circulation. Sulodexide treatment resulted in a decrease of the majority of the studied inflammatory parameters in both incompetent GSVs and systemic blood. The serum collected from the veins after sulodexide treatment caused lower levels of EC inflammatory markers as well as respective gene expression than the serum obtained before sulodexide treatment. Sulodexide application also reduced the inflammatory secretory activity of the senescent ECs.

Inflammation remains one of the most important factors in CVD development, and an increased level of inflammatory mediators has previously been documented in published studies.^2^^,^^3^^,^^24^ Previous studies have shown higher levels of vWF and IL-12 in the systemic circulation of varicose vein patients as compared with healthy subjects.^28^ Others have found significantly increased concentrations of IL-6, IL-8, and MCP-1 in the blood samples from the lower extremity from the varicose veins compared with the blood taken from the arm.^29^ In our study, we found that VCAM-1 levels were higher in serum samples collected from the incompetent GSVs as compared with the blood from the cubital arm veins. Higher levels of VCAM-1 present in the blood of the leg may reflect venous hypertension in patients with CVD. This observation was also documented in previous studies, together with the presence of increased plasma levels of endothelial leukocyte adhesion molecule-1 and intercellular adhesion molecule-1 in patients with CVD.^30^ In this study, the effects of sulodexide on IL-6, MMP-9, VCAM-1, and vWF were demonstrated in both the systemic circulation and lower extremity, leading to significant differential decreases in both the upper and lower extremities of patients with CVD. This observation suggests the possible impact of CVD not only on the LOL pathology but also on the potential systemic homeostasis abnormalities.^11^^,^^17^^,^^23^

In the in vitro part of this study, performed on the cultured ECs treated with the CVD sera, the exposure of the HUVECs to the blood serum from the incompetent GSVs obtained before sulodexide treatment resulted in significantly higher levels of MMP-9 concentrations in comparison with the effect of the exposure to the serum from UPL veins. The important role of MMP-9 in the CVD pathogenesis was investigated and emphasized in many studies evaluating the effect of MMPs on the vein wall degenerative changes as well as CVD-related skin pathology.^3^^,^^25^^,^^31^^,^^32^ The higher MMP-9 concentration after HUVEC exposure to the serum from LOLs was not followed by the similar observations concerning other inflammatory markers. Despite the elevated levels when compared with control, the levels of IL-6 and VCAM-1 did not differ when using the serum from systemic vs lower leg venous circulation. The reason for the lack of the above-mentioned changes after 24 hours of EC exposure to the CVD sera may in part be due to the short time of exposure to induce any significant changes in the cellular secretory activity. Another explanation is that the CVD serum before and after sulodexide treatment is performed on HUVECs that are normal, which may have a different behavior in the inflammatory expression compared with CVD ECs exposed to venous hypertension and constant circulating inflammatory mediators.^30^^,^^33^ The choice of the HUVEC model was based on the authors’ own experience and previous research where this cells were used for the evaluation of the effects of the serum collected from patients with various vascular pathologies including CVD.^24^^,^^34^, ^35^, ^36^

When studying the effect of the prolonged exposure to the CVD sera on EC senescence transformation compared with the control group, there was an increased detection of senescent markers expressed. The senescence of the ECs was more pronounced, as measured by prolongation of PDT, β-galactosidase activity, and p21 and p53 gene expression in ECs exposed to the serum from the incompetent GSVs as compared with the serum from the UPL of patients with CVD. The importance of p21 and p53 overexpression in CVD pathogenesis was also confirmed in other studies demonstrating that in the vein specimens taken from the proximal incompetent GSVs, there was an overexpression of p21 and p53 mRNA.^34^^,^^37^

Senescent ECs acquire the inflammatory and prothrombotic phenotype that can contribute to the pathology of CVD.^35^^,^^38^^,^^39^ As observed in this study, the serum from patients with CVD induced senescent cells with higher expression of genes regulating synthesis of IL-6, VCAM-1, MMP-9, and vWF compared with control. This reflects enhanced inflammatory and prothrombotic properties of ECs when subjected to the CVD serum. Previously, we found that the serum from patients with CVD accelerates the senescence of ECs.^35^ In this study, we demonstrated that the process of EC senescence is potentially more accelerated in the incompetent GSVs than the systemic venous circulation, which corresponded to the proinflammatory properties of the serum collected from the incompetent GSVs. The results of the study also suggest that anti-inflammatory intervention based on the medical treatment in patients with venous incompetence may prevent endothelial senescence transformation. Among the possible treatment modalities, sulodexide may be one of the potential options.^40^

Sulodexide is a drug with pleiotropic action in the intravascular space with an affinity for the endothelial layer and reducing the proinflammatory activation of ECs.^17^^,^^23^^,^^36^ In this study, the beneficial effect of sulodexide was confirmed in both the systemic venous circulation and the blood collected directly from the incompetent GSVs. Importantly, the intensity of both effects in the systemic and local venous circulation was comparable, indicating the potential protective effect of sulodexide on the studied inflammatory reaction-related factors. As demonstrated, sulodexide also reduced the CVD serum-induced proinflammatory and prothrombotic properties of the normal and senescent ECs. The sulodexide effects may be related to antioxidant effects, reduction in synthesis of inflammatory cytokines, cell autophagy, and stabilization of cellular DNA.^41^

Based on this study results, one can suggest that in patients with CVD-related EC senescence, significantly higher production of inflammatory mediators is found in the affected veins. Our results show that even without significant differences in the concentration of the inflammation markers (IL-9 and MMP-9) between the serum from the UPL and LOL veins, the serum from the LOL affected with CVD induced a significantly stronger EC senescence phenotype in the EC culture. However, as documented in this study, sulodexide is able to mitigate the EC inflammatory phenotype and reduce synthesis and release of cytokines, adhesion molecules, and prothrombotic factors. This latter finding has potential implications on the CVD pathophysiology and development, which should be clinically tested in future trials.^41^, ^42^, ^43^, ^44^, ^45^

Glycocalyx functional restoration is an important mechanism to prevent cardiovascular disease development, including atherosclerosis, diabetes, and CVD.^10^^,^^16^, ^17^, ^18^, ^19^, ^20^, ^21^^,^^46^ In our study, in the incompetent GSV biopsy specimens, the heterogenicity of the electron microscopy results concerning the glycocalyx thickness was observed with at least focally reduced thickness of this EC surface covering layer in patients with CVD. According to previously performed studies, the glycocalyx protection and restoration, together with inflammatory reaction downregulation and CVD symptom reduction, are important properties of sulodexide.^16^^,^^45^, ^46^, ^47^, ^48^ The limited number of GSV biopsies and especially the lack of the possibility to perform the same vein biopsy after 2 months of sulodexide treatment (providing glycosaminoglycan components) were the important limitations of our study, which did not allow us to confirm the final effect of the applied pharmacological therapy on the glycocalyx thickness. Among the other study limitations, the limited number of the recruited patients resulted in the lack of the possibility to assess the sulodexide clinical efficacy on the venous symptom reduction in this substudy group.

In conclusion, in patients with CVD, significant differences in the inflammatory markers between the systemic circulation and incompetent GSV segments can be observed. Systemic treatment with sulodexide reduces the proinflammatory conditions and senescent phenotype, as well as venous EC dysfunction. These findings may have potential clinical implications on CVD treatment. Further studies are needed to determine if sulodexide treatment can mitigate or reverse early disease (C2) advancement to higher clinical classes.

## Author Contributions

Conception and design: AZ, KJ, AB

Analysis and interpretation: AZ, AB, KK, MZ, TW, RK, JR, TU

Data collection: AZ, AB, KK, MZ, TW

Writing the article: AZ, AB, TU

Critical revision of the article: KJ, KK, MZ, TW, RK, JR

Final approval of the article: AZ, KJ, AB, KK, MZ, TW, RK, JR, TU

Statistical analysis: AZ, AB

Obtained funding: Not applicable

Overall responsibility: AZ

## Funding

The study was a part of research project funded by AlfaSigma Poland. AlfaSigma Poland involvement was limited to the coverage of the costs of the laboratory reagents and kits used in the laboratory assessment, but the company had no involvement in the collection, analysis, and interpretation of data. AlfaSigma Poland was not involved in the manuscript writing as well as in the decision to submit the manuscript for publication.

## Disclosures

A.Z. received funds for educative lectures supported by AlfaSigma Company. K.S.-J. is an employee of AlfaSigma Poland. R.A.K. was partly supported by a grant from the 10.13039/100000050National Heart, Lung, and Blood Institute, United States (HL147889-01A1). T.U. has been paid a consulting fee by AlfaSigma Company and is on their speakers’ bureau. The remaining authors report no conflicts.

## Figures and Tables

**Supplementary Table I (online only):** List of the primers

Gene	Forward primer	Reverse primer
p21	TGATTAGCAGCGGAACAAGG	TGCCAGGAAAGACAACTACT
p53	TTGCAATAGGTGTGCGTCAG	TCCCCACAACAAAACACCAG
IL-6	ATGCAATAACCACCCCTGAC	GAGGTGCCCATGCTACATTT
vWF	CCAGATTTGCCACTGTGATG	AAAGGCCTTCAGCACTTCAA
MMP-9	TCTATGGTCCTCGCCCTGAA	CATCGTCCACCGGACTCAAA
VCAM-1	GTTGAAGGATGCGGGAGTAT	TTCATGTTGGCTTTTCTTGC
GADPH	TTCGTCATGGGTGTGAACC	GATGATGTTCTGGAGAGCCC
HPRT	TGCTCGAGATGTGATGAAGG	TCCCCTGTTGACTGGTCATT

*GADPH,* Glyceraldehyde-3-phosphate dehydrogenase; *HPRT,* hypoxanthine phosphoribosyltransferase 1; *MMP-9,* matrix metalloproteinase-9; *VCAM-1,* vascular cell adhesion molecule-1; *vWF,* von Willebrand factor.

## Appendix

Additional material for this article may be found online at www.jvsvenous.org.
